# A new gene regulatory network model based on BP algorithm for interrogating differentially expressed genes of *Sea Urchin*

**DOI:** 10.1186/s40064-016-3526-1

**Published:** 2016-11-03

**Authors:** Longlong Liu, Tingting Zhao, Meng Ma, Yan Wang

**Affiliations:** 1 School of Mathematical Sciences, Ocean University of China, Qingdao, 266100 People’s Republic of China; 2 Key Laboratory of Mental Health, Institute of Psychology, Chinese Academy of Sciences, Beijing, 100101 People’s Republic of China

**Keywords:** BP algorithm, Gene regulatory network, Neural network model, Differentially expressed *Sea Urchin* genes

## Abstract

**Background:**

Computer science and mathematical theories are combined to analyze the complex interactions among genes, which are simplified to a network to establish a theoretical model for the analysis of the structure, module and dynamic properties. In contrast, traditional model of gene regulatory networks often lack an effective method for solving gene expression data because of high durational and spatial complexity. In this paper, we propose a new model for constructing gene regulatory networks using back propagation (BP) neural network based on predictive function and network topology.

**Results:**

Combined with complex nonlinear mapping and self-learning, the BP neural network was mapped into a complex network. Network characteristics were obtained from the parameters of the average path length, average clustering coefficient, average degree, modularity, and map’s density to simulate the real gene network by an artificial network. Through the statistical analysis and comparison of network parameters of *Sea Urchin* mRNA microarray data under different temperatures, the value of network parameters was observed. Differentially expressed *Sea Urchin* genes associated with temperature were determined by calculating the difference in the degree of each gene from different networks.

**Conclusion:**

The new model we developed is suitable to simulate gene regulatory network and has capability of determining differentially expressed genes.

## Background

Complex life phenomenon is the effect and regulatory mechanism of a large number of genes. To date, studies on complex biological systems have shifted from the local description of individual gene functions to quantitative analysis of complex gene regulatory networks (Plahte et al. 2013; Ahmad et al. 2012). Computer science and mathematical theory are combined to analyze the complex interactions among genes, which are simplified to a network to establish a theory model for the analysis of the structure, module and dynamic properties of a gene regulatory network (Smart et al. 2008; Patrik D’haeseleer SLaRS 1999; Raza and Parveen 2013).

From 2000, when the first published in nature on the topological properties of biological networks based on complex network theory to now, there is a huge development and progress achieved in the gene network investigation (Stifanelli et al. 2013; Bowers et al. 2004; Araki et al. 2013; Raza and Parveen 2013). Complex networks including the construction and simulation of a gene regulatory network are widely used in biological networks. Evidence from past data collection and statistical analysis of large-scale gene network highlights the compatibility of the structural characteristics of gene regulatory networks with other complex network system. Focusing solely on the network topology of a complicated network system is no longer sufficient in the process of constructing artificial gene networks.

The artificial network simulates the real gene network though network characteristics such as the average path length, clustering coefficient, average degree, modularity, map density and et al. (Thurner 2009; Raza 2016). It has been developed a variety of models and algorithms to simulate the gene regulatory network (GRN) mainly including the Boolean (Lähdesmäki 2003; Faure et al. 2006; Kim et al. 2007; Stolovitzky et al. 2008; Politano et al. 2014; Comar et al. 2015), Bayesian (Perrin et al. 2003; Husmeier 2003; Friedman et al. 2000; Bansal et al. 2006; Chai et al. 2014; Lo et al. 2015), linear differential equation (Chen et al. 1999; de Jong and Ropers 2006; van Someren et al. 2000), relevance (Butte and Kohane 2000; Runcie et al. 2012; Parmigiani et al. 2003) and neural network model (Vohradsky 2001; Rui et al. 2007; Raza and Alam 2016). However, traditional models of gene regulatory networks often lack an effective method of solving the gene expression profiling data because of high time and spatial complexity.

An artificial neural network (ANN) usually dubbed as “neural network” (the term we adopted and defined in this paper), is a computational model originally intended to simulate the structural and/or function of biological neural networks (Marshall 1995). And it exhibited powerful modeling ability and yielded significant results in terms of network structure, training algorithm, approximation performance, and stability (Aussem 1999; Mak et al. 1999). The use of recurrent neural network for constructing a gene regulatory network has achieved much better results than traditional models. However, the complexity of the recurrent neural network models makes it difficult and unsuitable for analysis of biologically significant gene regulatory relationships based on high-throughput microarray or sequencing data. Back propagation (BP) network as a kind of developed ANN is a multi-layered feed forward networks, in which the propagation is forward, error spreads reversely makes it faster and more powerful when used to model the high-throughput microarray or sequencing data than using the recurrent neural network algorithms.

Recently, reverse network model was developed as a suitable analysis for high-throughput data (such as microarray and high-throughput sequencing data) to mine regulatory mechanisms among the components of a system and has been extensively applied to examine various biological systems (Raza and Alam 2016; Werhli et al. 2006; Wang et al. 2010; Perkins et al. 2006). For increasing the accuracy of simulating GRN, we the first time mapped BP algorithm neural network based on sigmoid function into a common complex network with the microarray expression data. And thought the network parameters, the differential genes were determined. Rest of the paper is organized as follows. In method part, BP network was described briefly and the genes networks based on BP ANN was built. The result part is model application and comparison. Then discussions were presented and finally paper was concluded.

## Methods

Reverse network model is built based on BP network combined with complex nonlinear mapping and self-learning. An artificial network is simulated the real gene network according to the network characteristics: the average path length, average clustering coefficient, average degree, modularity, map’s density and et al.

### Structure and algorithm of the BP neural network

**Fig. 1:**
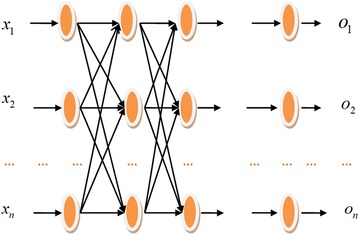
Structure chart of the feed forward neural network

The neural network is a computational model which originally was used in the simulation of the structure of biological neural network and used for other computational simulations lately, for example, evaluating the landslide susceptibility and predicting the liver injury (Sukumar et al. 2012; Rampone and Valente 2011). The algorithm of BP network we adopt in this paper has already been detailed before (Rampone and Valente 2011; Cao et al. 2016; Liu et al. 2016). The classical artificial neural network structure is a feed forward network (Fig. 1) with multiple layers consists of an input layer, an output layer and a hidden layer with different roles. Each neuron of a given layer is connected to all the neurons of the next one and each connecting line has an associated weight. There are three procedures to build the whole structure with three equations. First, neuron receives the weighted sum of the input patterns and/or of the other neuron outputs as an input.

1 $$o_{k} = f\left( {\sum\nolimits_{n} {w_{kn} o_{n} - b_{k} } } \right)$$

*w*_*kn*_: The weight from neuron *n* to neuron *k*; *o*_*n*_: the output of neuron *n* or of the nth input: *b*_*k*_: the neuron threshold.

The transfer function in our experiment is chosen the sigmoid function:

2 $$f({\text{x}}) = 1/(1 + {\text{e}}^{ - x} )$$

The training procedure adopted BP algorithm. During the training the weights and biases of the network are iteratively adjusted to minimize the difference (error) when the output value isn’t equal to or less than the desired output, until the mean square error (MSE) of the system is minimized. The error *E* ^*p*^ of a given the pth pattern is calculated as

3 $$E^{p} = 1/2 \times \sum\limits_{j} {\left( {t_{j}^{P} - o_{j}^{P} } \right)}^{2}$$

*t*_*j*_^*p*^: the pth desired output value; *o*_*j*_^*p*^: the output of the corresponding neuron.

The rule of the error BP algorithm used in this study as following.

- Initialization: small random values are taken for weights of each layer and thresholds of each neuron, the max cycle times and the min whole error are set as *m* and *ɛ* respectively;
- Vector $X^{{^{p} }} = \left[ {x_{0}^{P} ,x_{1}^{P} , \ldots ,x_{n - 1}^{P} } \right]$ is the inputted data pattern, where P means the Pth pattern, and *n* is the number of neurons of initial layer, *x*_*i*_^*P*^ is the input of the given hidden layer;
- The actual output of hidden layer $O_{j}^{p} = f\left( {\sum\nolimits_{j = 0}^{n - 1} {w_{ij} x_{ji} - B_{i} } } \right)$ is calculated and regarded as an input to the next layer, *f* is the activation function;
- If the layer is the last layer (output layer), the actual error *E* ^*P*^ is calculated as $E^{P} = \sum _{j} \left( {t_{j}^{P} - o_{j}^{P} } \right)^{ 2}$, otherwise the error calculation as (c);
- The whole error *E* is calculated as $E = 1/ 2 \times \sum\limits_{{_{P} }} {E^{P} }$;
- Weights are adjusted from the last layer and going backwards (BP),
  4 $$W_{ji} ({\text{new}}) = W_{ji} ({\text{old}}) + \eta \times \delta_{j}^{p} o_{j}^{p} +\upalpha \times \text{(}W_{ji} ({\text{new}}) - W_{ji} ({\text{old}})\text{)}$$
  where *η* (0 < *η* < 1) and *α* (0 < *α* < 1) are constants named learning rate and momentum, respectively; *η* measures the influence degree of the error; *α* determines the influence of the weight change.
  When neuron *j* is output layer neuron and hidden layer neuron, the error term for pattern *p* is $\delta_{j}^{p} = f^{\prime }\left( {{\text{o}}_{j}^{p} } \right)\left( {{\text{t}}_{j}^{p} - {\text{o}}_{j}^{p} } \right)$ and $\delta_{j}^{p} = f^{\prime }\left( {{\text{o}}_{j}^{p} } \right)\left( {\sum\nolimits_{k} {W_{kj} \times \delta_{k}^{p} } } \right)$ respectively;
- If the cycle time is *m* or the whole error is less than *ɛ*, train is over, otherwise go to (b).

The weight adjusting in the above algorithm is aimed at minimizing the whole error *E*, which is performed with the gradient descent via weight changing to make the error steepest down (Bishop 1996). The *η* term is a measure of the influence degree for updating weights in the formula, whereas the *α* term determines the influence of the past history of weight changes in the same formula. The single-layer neural network structure is a two-layer network structure with input and output layer. It facilitates more easily the mapping from the trained BP network to the gene regulatory network, so we will use the single-layer BP network in this paper.

### Establishment of genes networks based on BP ANN

**Fig. 2:**
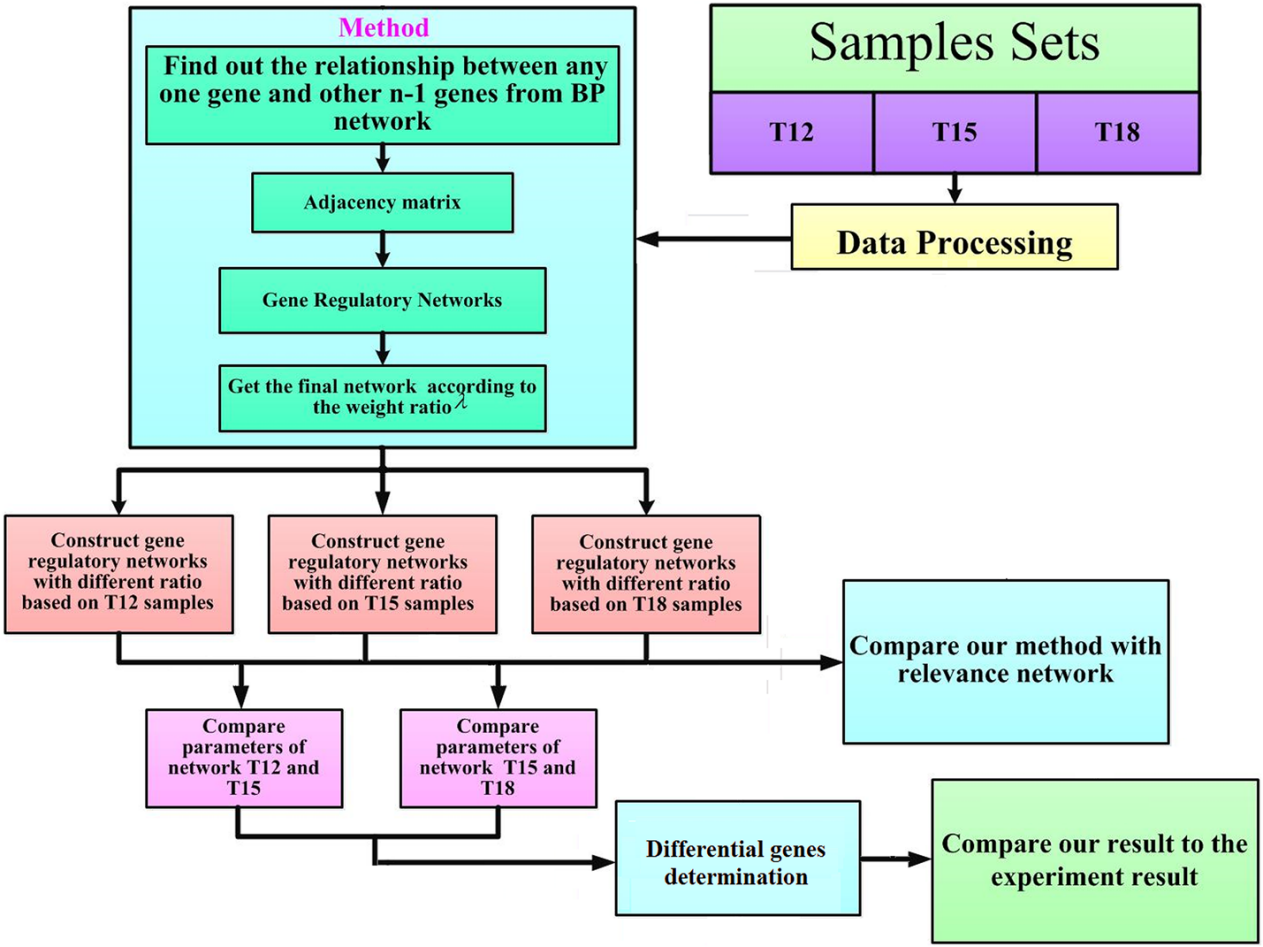
The flowchart of model architecture and the structure of the paper. The model takes microarray data as input, and will be trained as described in flowchart: finding out the relationship between any one gene and other n − 1 genes, making adjacency matrix, building gene regulatory network and getting the final gene network according to the weight ratio λ. The training is carried on in each group respectively. The network is compared with the common relevant network by the value of parameters and the differential genes determined by the network are compared with that determined by fold_change

The architecture diagram of the proposed model is shown in Fig. 2. The model takes microarray data as input, and will be trained as described in flowchart: finding out the relationship between any one gene and other n − 1 genes, making adjacency matrix, building gene regulatory network and getting the final gene network according to the weight ratio λ. The training is carried on in each group respectively.

In the network, the gene is simplified as a node, the regulation is simplified as the connection between nodes (edge), and the gene regulatory network is composed of nodes set V and the set of edges between nodes in *E*:

$$G = \, \left( {V, \, E} \right)$$

Given that the adjacency matrix can be used to describe the relationships between nodes in a network, the topology of the network is represented by adjacency matrix *A*:

$$A_{n \times n} = \;\left[ {\begin{array}{*{20}c} 0 &\quad {a_{21} } &\quad \cdots &\quad {a_{1n} } \\ {a_{12} } &\quad 0 &\quad \cdots &\quad {a_{2n} } \\ \cdots &\quad \cdots &\quad \cdots &\quad \cdots \\ {a_{n1} } &\quad {a_{n1} } &\quad \cdots &\quad 0 \\ \end{array} } \right]$$

where *a*_*ij*_ and *a*_*ji*_ in *A* represents the regulation between gene *i* and *j*. Assuming that the state changes of genes in a real regulatory network mainly depend on the effect of other genes, the self-regulation of a gene can be ignored. Then, the diagonal elements in *A* are 0, i.e. *a*_*ij*_ = *0*. Two kinds of regulatory relationships exist between genes, activation and inhibition which indicated by the negative or positive value of *a*_*ij*_.

**Fig. 3:**
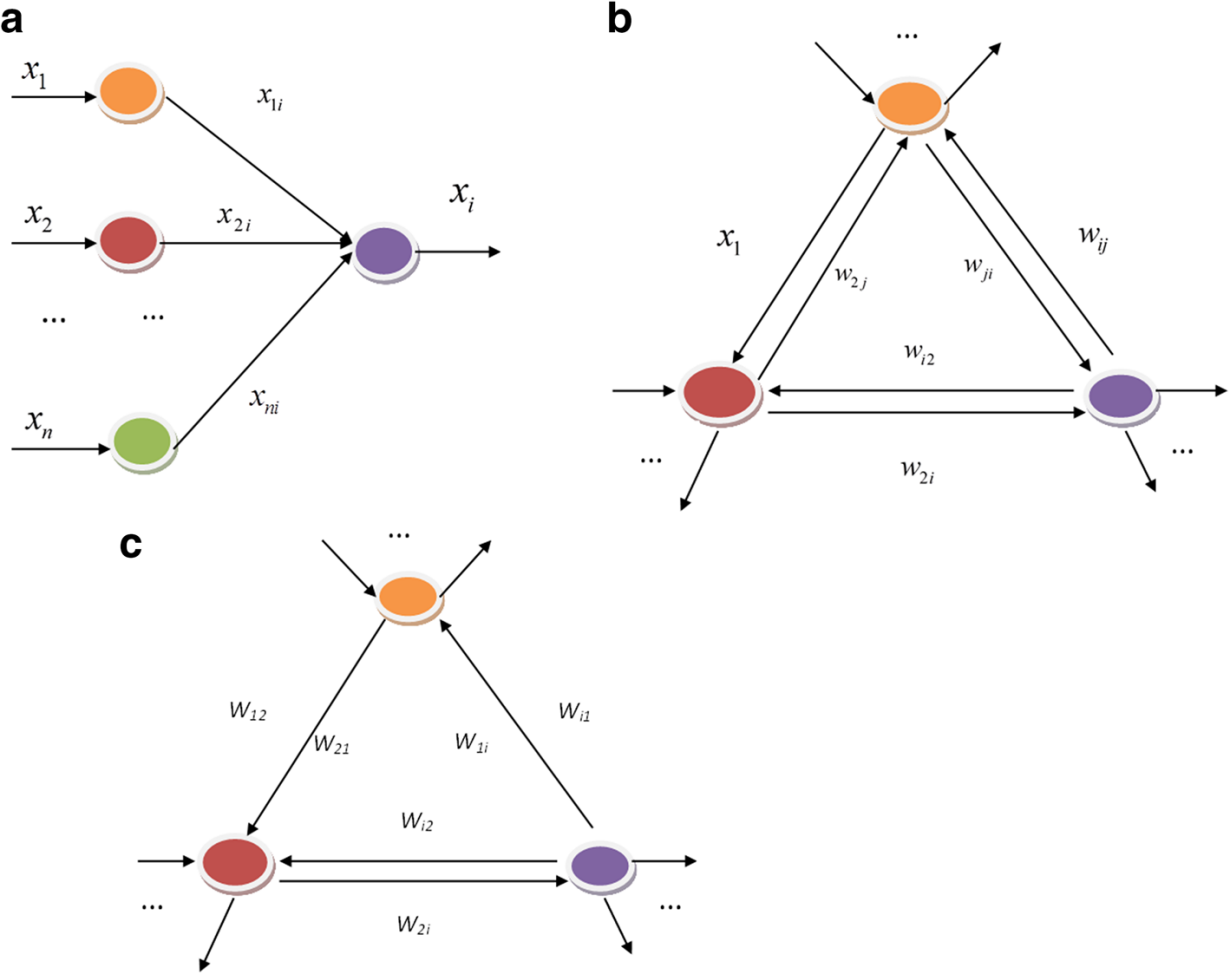
Structure chart of **a** linear neural network, **b** initial gene regulatory network and **c** final gene regulatory network

First, a single-layer BP neural network with *(n* − 1*)* − 1 structure is adopted to construct the model (Fig. 3a). The *n* − 1 neurons in the input layer are used as the temporary storage for the input data corresponding to the *n* − 1 genes. The neuron in the output layer is corresponding to the *n*th genes. Self-correlation is not considered in this model which means network weight *w*_*ii*_ doesn^’^t exist. So, the input samples for the network are $X^{p} = x_{1}^{p} , \cdots ,x_{n - 1}^{p}$, and the target sample is $T^{p} = x_{1}^{p}$. The training samples are {*X* ^*p*^, T^*p*^}, i.e., the input samples are the same as the target samples. The *p* training samples are inputted to the network to train the network until the error or the operating cycle reaches the set value. Therefore, a weight vector *W*_*i*_ can be obtained. This process is reiterated form *x*_1_ to *x*_*n*_, and then, a weight matrix *W* is obtained.

5 $$X = F\left( {{\text{W}}^{T} {\text{X}}} \right)$$

$$\left[ \begin{aligned} o_{1} \hfill \\ o_{2} \hfill \\ \cdots \hfill \\ o_{n} \hfill \\ \end{aligned} \right] = F\left( {\left[ {\begin{array}{*{20}c} 0 &\quad {w_{12} } &\quad \cdots &\quad {w_{1n} } \\ {w_{21} } &\quad 0 &\quad \cdots &\quad {w_{2n} } \\ \cdots &\quad \cdots &\quad \cdots &\quad \cdots \\ {w_{n1} } &\quad {w_{n2} } &\quad \cdots &\quad 0 \\ \end{array} } \right]\left[ \begin{aligned} x_{1} \hfill \\ x_{2} \hfill \\ \cdots \hfill \\ x_{n} \hfill \\ \end{aligned} \right]} \right)$$

$o_{i} = f\left( {w_{1i} {\text{x}}_{1} + \cdots w_{ij} {\text{x}}_{j} + \cdots w_{ni} {\text{x}}_{n} } \right),{\text{j}}\;{ \ne }\;{\text{i}} .$ When the training error is very small [Eq. (4), *E* < 10^−2^], *o*_i ≈_ *x*_*i*_ can be considered; That is, any gene among the *n* genes can become a sigmoid function of the linear combination of the other *n* − 1 genes.

Second, the trained BP neural network is mapped into a gene regulatory network. The trained neural network is mapped into a directed gene regulatory network (Fig. 3b). *A* = *W*, weight $w_{ij} \left( {{\text{i}}\;{ \ne }\;{\text{j}}} \right)$ denotes the edge weight from neuron *i* to neuron *j* in the gene regulatory network, whereas $w_{ji} \left( {{\text{i}}\,{ \ne }\,{\text{j}}} \right)$ denotes the edge weight from neuron *j* to neuron *i*.

Third, a reasonable weight threshold is selected to choose the highly relevant genes and finally determine the gene regulatory network. The process of choosing threshold is very important. The threshold can be set according to the weight ratio $\lambda = {{\left| {w_{ij} } \right|} \mathord{\left/ {\vphantom {{\left| {w_{ij} } \right|} {\sum\nolimits_{i} {\left| {w_{ij} } \right|} }}} \right. \kern-0pt} {\sum\nolimits_{i} {\left| {w_{ij} } \right|} }}$ or by other methods. In the regulatory network, the edges with weight ratio which is less than the threshold are removed to obtain the final gene regulatory network. The weight ratios corresponding to weight *w*_1i_ and *w*_21_ are assumed to be less than the threshold and are deleted, and then the corresponding edges in the regulatory network are also removed (Fig. 3c).

### Structural parameters of network

The network statistics used to describe the network structure are briefly explained in this section. *G* = *(V, E)* is assumed to be a complex network with node set *V* = *{1,2,…N}* and edge set *E*. The parameters are determined according to the network statistics as following:

1. Average path length (*L*)

The distance *d*_*ij*_ between nodes *i* and *j* in *V* is defined as the minimal number of edges connecting nodes *i* and *j*. The average path length of the network is defined as $L = \sum\nolimits_{i > j} {{{d_{ij} }/ {\left( {0.5{\text{N(N}} - 1)} \right)}}}$.

2. Average clustering coefficient (*C*)

The clustering coefficient *C*_*i*_ of node *I* is the ratio of the number of actually existing edges to that of possible existing edges among the adjacent nodes of *i*. The clustering coefficient of the network *C* is the average of the clustering coefficients of all nodes.

3. Average degree (*K*)

The degree of a node is the number of other nodes connecting to this node. The average degree *K* of the network is the average of degrees of all nodes.

4. Modularity (*Q*)

Network *G* is supposed to contain *k* communities as G_1_, G_2_, …G_k_. A symmetric matrix *H* = (h_*ij*_)_*k* × *k*_ is defined, where *h*_*ij*_ denotes the ratio of the number of edges between two communities *G*_*i*_ and *G*_*j*_ to the number of total edges of the network.

Modularity is defined as

$$Q = \sum\nolimits_{i} {Q_{i} } = \sum\nolimits_{i} {\left( {h_{ij} - \alpha_{ij}^{2} } \right)}.$$

where *α*_*i*_ denotes the sum of elements in the *i*th row of matrix *H*, which represents the ratio of the number of edges connecting to community *G*_*i*_ to the number of total edges.

5. Density of map (*D*)

Density of map is the ratio of the total path length to the area of the map.

## Results

In this section, we applied the network model on the microarray data to determine the differentially expressed genes and to assess how the model works. We chose the *sea urchin* (*Strongylocentrotus purpuratus*) mRNA microarray data in the GPL13644 platform from NCBI database in our analysis (http://www.ncbi.nlm.nih.gov/geo/query/acc.cgi?acc=GPL13644).

### Data sources

In this experiment, mRNA microarray data of *Sea Urchin* was used to investigate if the gene expression responses characterize molecular signatures of temperature stress, and as a result to know how stress responses alter gene expression. There were totally 191 samples divided into three groups according to the different temperature: 64 samples at 12 °C (T12), 63 samples at 15 °C (T15), and 64 samples at 18 °C (T18), respectively (Runcie et al. 2012). We applied the microarray data, 336 transcripts totally, to the network model to build gene networks and to analyze what genes responding to different growing temperature stress are significantly differential expressed.

### Data processing

To remove the impact of the differences of the original gene records on the model, each gene record is normalized to [0, 1] using the following formula:

1. If $$x_{\rm min}\,{\ne}\,x_{\rm max}$$, then $$x^\prime\,=\,\frac{x\,-\,x_{\rm min}}{{x_{\rm max}\,-\,x_{\rm min}}}$$;
2. If $$x_{\rm min} = x_{\rm max}$$, then $$x^{\prime } = x_{\rm min}$$.

where, *x* represents the element of each sample. *x*_min_ and *x*_max_ represent the minimum and maximum of all the samples elements, respectively, and *x*′ represents the normalized sample element.

To further reduce the noise from the different experimental conditions of the samples, the samples of each group were divided into two different blocks and the mean value of each sample was computed. So, a total of 32, 31, and 32 samples were observed in the T12, T15, and T18 groups.

### Establishment of gene networks

The model of a single-layer feed forward neural network with the structure of 335-1 was shown in Fig. 3a. The 335 neurons in the input layer (temporary storage) correspond to the data of 335 genes, whereas the neuron in the output layer corresponds to the data of another gene of the 335 genes. Therefore, the input sample of the network is $X^{p} = x_{1}^{p} , \ldots ,x_{335}^{p}$, the target sample is $T^{p} = x_{i}^{p}$, and the training sample is {*X* ^*p*^, T^*p*^}. The transfer function of neurons is sigmoid function with learning rate 0.7 and the threshold −1.

**Fig. 4:**
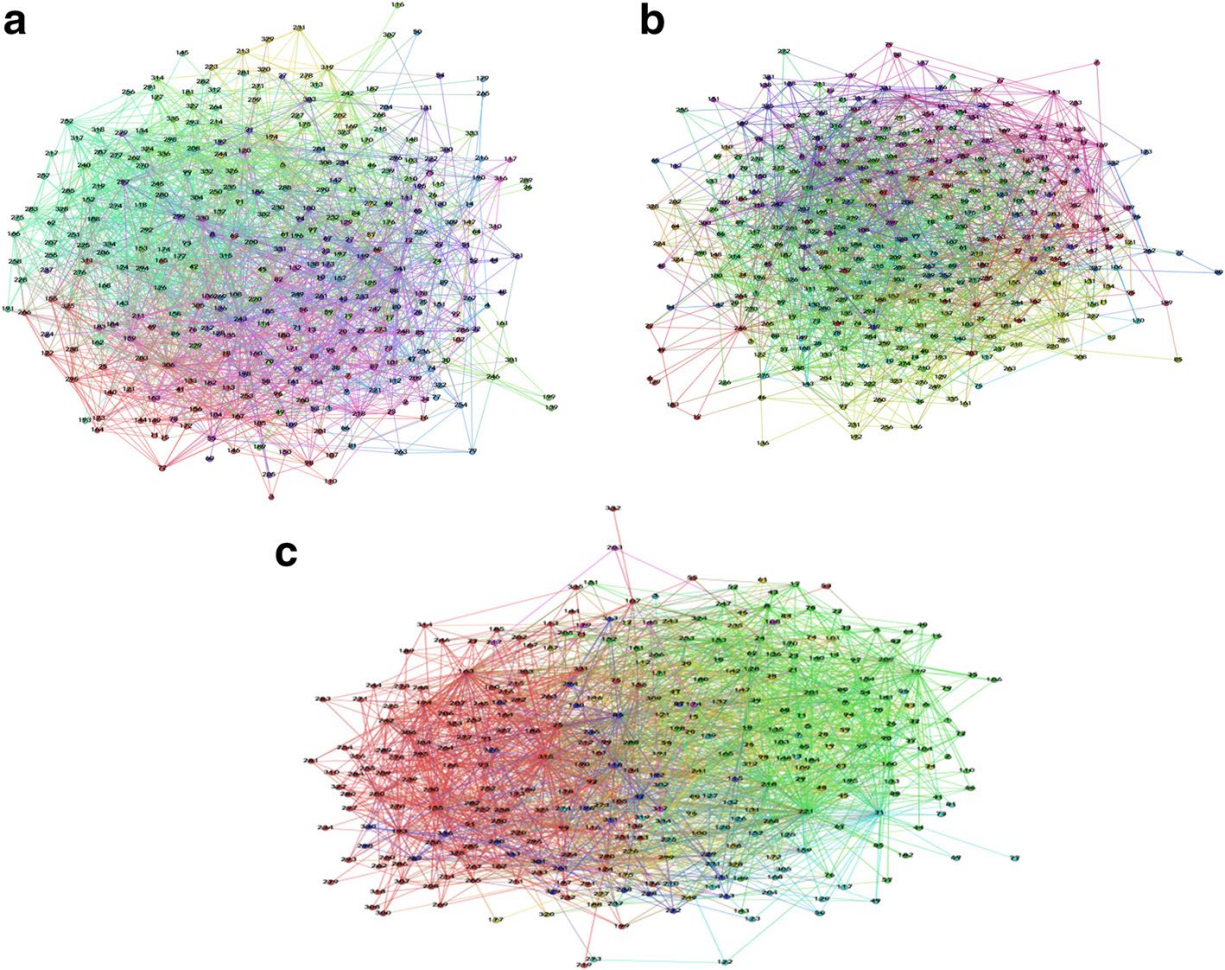
Structure chart of the networks with the weight ratio of 0.85 based on **a** T12 group, **b** T15 group and **c** T18 group

First, the 32 normal samples of the T12 group are considered as the training set to train the network until the error or operating cycle reaches the set value (the initial values of all weights are set to be identical for comparability). Then trained BP neural network is mapped into a gene regulatory network. There were 10 different weight thresholds with weight ratio λ of 0.5, 0.55, 0.6, 0.65, 0.7, 0.75, 0.8, 0.85, 0.9, and 0.95 selected to construct 10 gene regulatory networks with different relevance, respectively (Fig. 4a). Finally, the parameters of the 10 gene regulatory networks are counted (according to the given five parameters we mentioned). The samples from the T15 and T18 are subjected to the same treatment (Table 1; Fig. 4b ,c).

**Table 1:** The parameters of 3 networks constructed based on samples of T12, T15 and T18 group, respectively

Samples	Average path length (*L*)	Average clustering coefficient (*C)*	Average degree (*K*)	Modularity (*Q*)	Density of map(*D*)
T12	3.74	0.082	6.875	0.273	0.021
T15	3.833	0.049	5.765	0.287	0.017
T18	3.256	0.109	7.426	0.264	0.022

### Comparison of gene networks

**Fig. 5:**
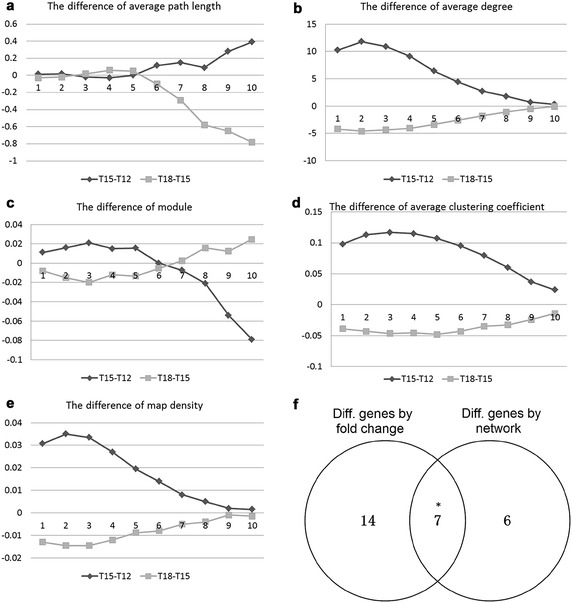
Difference of the different parameters and comparison of differential genes. Parameters of** a** average degree, **b** average Path, **c** modularity, **d** average clustering coefficient and **e** map’s density; **f** Venn diagram between differential genes determined by network and fold_change

The parameters of the three networks (10 each) constructed based on the samples from T12, T15, and T18 are compared. Table 1 presents the parameters of the gene regulatory networks from different time groups with weight ratio 0.85. All the parameters of the three networks we compared are different from each other. To further clarify the differences of three networks, the parameters of T12 and T18 networks are compared to T15 in different weight ratios as showed in Fig. 2. The horizontal axes represent the weight ratios; ten different weight ratios were increase distributed from 0.5 to 0.95 (0.5, 0.55, 0.6, 0.65, 0.7, 0.75, 0.8, 0.85, 0.9, and 0.95) (Fig. 5a–e). The vertical axe represents the value of the parameters in different figures. From Fig. 5a–e are the average degree, average path length, modularity, average clustering coefficient and density of map, respectively. The light gray lines in the figures denote the differences of the network parameters between the training samples of T18 and T15, whereas the dark gray lines denote the parameter difference between T15 and T12. Evident differences are observed in different weights and in the different parameters. The smallest differences in the parameters of the average path lengths and modularity are nearly at the weight ratio of 0.75, while the smallest differences of other three parameters are at the weight ratio of 0.95. The differences indicate that difference group has its suitable weight ratio and also proved the effectiveness of the model.

To compare the new model with the common relevance network, the same samples were used to construct the relevance network based on Pearson’s coefficient. Table 2 shows the parameters of relevance network with correlation coefficient 0.85. Compared with the parameters with weight ratio 0.85 based on BP algorithm (Table 1), parameters of average clustering coefficient and density of the map based on the relevance network are zero, and parameter of average degree is close to zero, much lower that from BP algorithm. There are 113, 130 and 180 genes in T12, T15 and T18 groups with zero degree in relevance network. Evidently, it’s not reasonable that more than one-third genes with zero degree and moreover that the zero degree of average clustering coefficient and map’s density in all three groups from the common relevant network makes parameters no sense and consequently decrease the accuracy. So BP neural network tends to be more suitable for reconstructing the network than the relevance network based on Pearson’s coefficient.

**Table 2:** Parameters of relevance network with correlation coefficient 0.85

Samples	Average path length (*L*)	Average clustering coefficient (*C*)	Average degree (*K*)	Modularity (*Q*)	Density of map (*D*)
T12	1	0	0.006	0	0
T15	1.167	0	0.03	0.72	0
T18	1	0	0.024	0.75	0

### Differentially expressed genes determination

Another distinguishing function of our model is differentially expressed genes determination, in which *η* was introduced and defined as degree difference ratio:

$$\eta = \frac{{\left| {T_{i} - T_{i}^{\prime }} \right| + \left| {T_{0} - T_{0}^{\prime }} \right|}}{{\left( {T_{i} + T_{0} } \right) + \left( {T_{i}^{\prime }+ T_{0}^{\prime }} \right)}}$$

where *T*_i_ and *T*_0_ denote the input and output degrees of a gene in the network of one group; $T_{i}^{{\prime }}$ and $T_{0}^{{\prime }}$ denote the input and output degrees of a gene in the network of another group.

The gene regulatory networks are constructed based on the samples from the T12, T15, and T18 groups with weight ratio λ of 0.85. The degree difference ratio *η* of each gene is calculated using samples from T15 and T12 group. The genes are ranked according to *η* in descending order. And the same process is performed using samples from T18 and T15 groups. In this experiment, 13 differentially expressed genes are found (Table 3). At the same time, we calculate the significantly differential expressed genes (DEGs) using the same data after normalization and outlier removal. The significantly differential genes are defined as |log2FC| ≥ 0.05 and p value <0.05 by z-test. There are 22 DEGs in common between T12 and T18 compared with T15 (Two groups of differential genes were calculated between T12 and T15, and between T18 and T15, the same as network.). Comparison is carried on between DEGs calculated by BP algorithm and by fold change (Fig. 5f). There were 7 DEGs (*APOBEC,FoxG,FoxO,gataC,Gsk*-*3,OTX,SM30*-*E*) significantly overlapped indicating that the BP network model we build has capability of finding a large part of the significantly differential genes based on the high-throughput sequencing data or microarray data determined by experimental method.

**Table 3:** List of differentially expressed genes

ID	ORF	SEQUENCE
1776	APOBEC	ATAAGAACCAGTGGGGCCCACCCAGTTTCACCCTCCTCTCTCAT
5123	Otp	ACCCGCATCGCAATCTCCTCCCGCATGAAGATATCAGGATAGT
2789	Gsk-3	GTCCTAGGAACCCCAAGCCGTGACCAGATCAAGGAGATGAAC
1078	Nk1	GCCATCATCACCCGACCCAACTGCAGCAGCTATTCATACAT
6021	gataC	TAGTTCAGCACCTCATCCCGGTCCAACAAGTTCCTACACGTTACC
3972	FoxG	TCATGATGGCTATTCGCTCGAGTCCAGAGAAAAGACTAACTCTAAATG
4071	G-cadherin	GTGCGAGGAGACCAGCCTTTCCATCGAGTTCATCACAGAGACTC
5397	Blimp1-Krox	ACCTATGTATGGCCTGTCACCAAACTACATCAGTACTGCAGGTGGT
3498	FoxO	CGATCATGACCACACATCCAGAAATCGACATGCATGACAATGAAGTC
265	APOBEC	ACAACAGCTCCTCCCCTCACCCCTACCAGTCAGGCTACCACC
2208	SM30-E	TCAACCTGGTTTTGGACAACCCGGTGTTGGTCAACCCAATAGA
5073	otx	GCACTTTCTGATCTTGCTAGTCGTGAAATCAAGATGGAATCACATTCT
1987	P16	AAGTGATGACGACGGCAGCAGCGATGATGACGGTAGCAGTGAT

## Discussion

To date, most gene regulatory networks are small networks for hundreds of genes. Traditional models of gene regulatory networks often lack an effective method of solving gene expression profiling data because of high time and space complexity. Based on predictive function construction and network topology, a new model for constructing gene regulatory networks using a BP neural network was tested in this paper. Combined with complex nonlinear mapping and self-learning, the BP neural network was mapped into a complex network. Since ANN can easily implement parallel processing, building a large-scale gene regulatory network model with different layers and modules is possible. Concretely, the internal characteristics and operation mechanism of the function modules of the network should be investigated. And the function and robustness under the outside interference of a sub-network should be discussed according to the classification of a regulatory network structure as well.

Mathematical theory has shown that multilayer feed forward BP networks can carry on any complex nonlinear functions, making it particularly suitable for solving problems with complex internal mechanism. BP networks have the ability of self-learning and generalization, but are also limited by slow learning speed, difficulty in determining the number of hidden layer nodes, and falling into local minima. In this study, we adopt a single-layer network structure, in which there is no hidden layer. Thus, selection of initial values of the network parameters is performed more frequently and the local minima are more likely avoided. Without changing the network structure, the data is added to the training set directly. With the training of the neural networks, network weights and the mapped gene regulatory networks are changed.

Through statistical analysis and comparison of differential genes based on the mRNA microarray data from *Sea Urchin* growing in different temperatures, parameters of diverse average degrees, average path lengths, modularity, average clustering coefficients, and map densities were obtained. Differentially expressed *Sea Urchin* genes associated with temperature were determined by calculating the difference in the degree of each gene from different networks. To check the effectiveness of BP network, comparison of the parameters with the common relevance network based on Pearson’s coefficient and significantly overlapped differential genes showed that the parameters of BP network were more efficient to build gene regulatory network. The remain un-overlapped genes reminded us that the gene regulatory network built based on BP network still need to improve maybe though improving some algorithm.

Besides, the convergence of a network is important that reducing, maintaining, or increasing the training error of the network within a specific controlled range allows the retention of newly added samples in the training set by the convergence of a network and should be ensured. If the error is large, the sample is regarded as a singular point and cannot be retained in the training set. Therefore, the dynamic property and stability of the network should be guaranteed.

## Conclusion

In this paper, we developed a new model for constructing gene regulatory networks based on back propagation neural network. The application of the new model to the mRNA microarray data and the comparison with the common reverse network and differential genes indicated that the new model is suitable to simulate gene regulatory network and has capability of determining differentially expressed genes.
